# Randomized clinical trial analyzing maintenance of peripheral venous catheters in an internal medicine unit: Heparin vs. saline

**DOI:** 10.1371/journal.pone.0226251

**Published:** 2020-01-06

**Authors:** María Jesús Pérez-Granda, Emilio Bouza, Blanca Pinilla, Raquel Cruces, Ariana González, Jesús Millán, María Guembe

**Affiliations:** 1 Department of Clinical Microbiology and Infectious Diseases, H.G.U. Gregorio Marañón, Madrid, Spain; 2 Department of Nursing, School of Nursing, Physiotherapy and Podiatry, Universidad Complutense de Madrid, Madrid, Spain; 3 CIBER Enfermedades Respiratorias-CIBERES (CB06/06/0058), Madrid, Spain; 4 Medicine Department, School of Medicine, Universidad Complutense de Madrid, Madrid, Spain; 5 Infection Study Group of the Sociedad Española de Medicina Interna, Madrid, Spain; 6 Department of Internal Medicine, H.G.U. Gregorio Marañón, Madrid, Spain; 7 Instituto de Investigación Sanitaria Gregorio Marañón, Madrid, Spain; Universite de Bretagne Occidentale, FRANCE

## Abstract

**Background:**

Peripheral venous catheters (PVCs) require adequate maintenance based on heparin or saline locks in order to prevent complications. Heparin has proven effective in central venous catheters, although its use in PVCs remains controversial. Our hypothesis was that saline locks are as effective as heparin locks in preventing problems with PVCs. The objective of the present study was to compare phlebitis and catheter tip colonization rates between PVCs locked with saline and those locked with heparin in patients admitted to an internal medicine department (IMD).

**Methods:**

We performed a 19-month prospective, controlled, open-label, randomized clinical study of patients with at least 1 PVC admitted to the IMD of our hospital. The patients were randomized to receive saline solution (PosiFlush^®^, group A) or heparin (Fibrilin^®^, group B) for daily maintenance of the PVC. Clinical and microbiological data were monitored to investigate the frequency of phlebitis, catheter tip colonization, and catheter-related bloodstream infection (C-RBSI), as well as crude mortality, days of hospital stay, and days of antimicrobial treatment.

**Results:**

We assessed 339 PVCs (241 patients), of which 192 (56.6%) were locked with saline (group A) and 147 (43.4%) with heparin (group B). The main demographic characteristics of the patients were distributed equally between the 2 study groups. The median (IQR) catheter days was 5 (3–8) for both groups (p = 0.64). The frequency of phlebitis was 17.7% for group A and 13.3% for group B (p = 0.30). The frequency of colonization of PVC tips was 14.6% and 12.2% in groups A and B, respectively (p = 0.63). Only 2 episodes of C-RBSI were detected (1 patient in group A). Saline lock was not an independent factor for phlebitis or catheter colonization.

**Conclusions:**

Our study revealed no statistically significant differences in the frequency of phlebitis and catheter tip colonization between PVCs locked with saline and PVCs locked with heparin. We suggest that PVC can be maintained with saline solution, as it is safer and cheaper than heparin.

## Background

Intravascular catheters play an indispensable role in patient management. Peripheral venous catheters (PVC) are used increasingly in hospitals [1–4].

Proper maintenance of PVCs is essential if we are to prevent phlebitis, obstructions, and bacteremia [5–7]. A study review found that 10% (4,204) of 40,620 PVCs in 51 countries presented phlebitis [4].

A study in different departments of internal medicine in Spain collected phlebitis rates of 3.8–5.1% [8]

Maintenance requires periodic catheter locks with either heparin or saline in order to prevent complications. Heparin has proven efficacious specially in short term central venous catheters, which was slightly superior to saline for catheter locks [9]. However, its usefulness in PVCs remains controversial [10–16].

We compared the efficacy of saline and heparin locks in PVC maintenance in terms of phlebitis and catheter tip colonization rates.

## Material and methods

### Setting

Our institution is a general reference teaching hospital with 1,550 beds and approximately 55,000 admissions/year. Our Internal Medicine Department (IMD) is a 30-bed unit.

### Study design

We performed a prospective, randomized clinical trial over a period of 19 months (October 2015-October 2017). The authors confirm that all ongoing and related trials for this intervention are registered.

The study population comprised patients admitted to the IMD with at least 1 PVC. The patients who gave their informed consent were randomly assigned to 2 groups (1:1): saline (PosiFlush^®^, group A) or heparin (Fibrilin^®^, group B). Randomization was performed by the principal investigator of this project. A computer generates random number table was used to assign the groups. The envelopes were prepared by the principal investigator and consecutive numbered envelopes were generated containing the protocol and the assigned group to which patients would be included. Two groups of patients were chosen by a random procedure.

As the catheters of each patient were locked according to the group to which they belonged, the primary outcome measure was analyzed at catheter level, not at patient level. Therefore, the groups were not the same, as a patient can not have two catheters and that each catheter belonged to a different randomization group.

### Inclusion and exclusion criteria

The inclusion criteria were age ≥18 years, no evidence or suspicion of C-RBSI at enrolment, no history of allergy or intolerance to heparin, and no coagulation abnormalities.

The exclusion criteria were catheters inserted more than 24 hours.

### Study medication

#### Catheters with medication ≤24 hours

Group A: were flushed with 3 cc of saline (PosiFlush^®^) once per shift (each 8 hours).

Group B: were flushed with 60 units of heparin (Fibrilin^®^) once per day. Saline was flushed before it´s use according to the manufacturer´s recommendations.

#### Catheters with medication ≥24 hours

Group A: were flushed with 3 cc of saline after each use.

Group B: were flushed with 60 units of heparin after each infusion. Saline was flushed before it´s use according to the manufacturer’s recommendations.

#### Catheter maintenance

Catheter care included the following: daily recording of the need for catheter use, daily monitoring of the insertion site, skin disinfection with 2% alcoholic chlorhexidine, connector disinfection with 70% alcohol wipes before use, hand hygiene, replacement of gauze/transparent dressing according to international guidelines, and use of split-septum closed connectors (CLAVE, ICU Medical, Inc., San Clemente, CA, USA) [17] [18–20]. PVCs were daily monitored by the nurse who cared them, and information was collected in the patient records.

Withdrawn catheters were included in the study and considered to belong to the group to which they had previously been randomized.

Patients were followed up until the catheter was removed or until discharge.

All catheters were withdrawn when clinically required, and the catheter tips, needleless connectors, and superficial cultures (skin and hub) were systematically sent for culture.

The study was registered at www.clinicaltrials.gov (NCT02970409).

### Endpoints

The primary endpoint was the frequency of phlebitis and/or catheter tip colonization rates in PVCs locked with either saline or heparin in patients admitted to the IMD.

The secondary endpoints were C-RBSI rate, catheter obstruction rate, indwelling time (days from insertion to withdrawal), adverse effects associated to heparin use such as bleeding or heparin-induced thrombocytopenia, hospital stay, and mortality rate.

#### Definitions

Phlebitis. Presence of 1 or more of the following criteria: swelling and erythema >4 mm, tenderness, palpable venous cord, and pain or fever with local symptoms. Isolated swelling was not defined as phlebitis.

Catheter tip colonization. Isolation of either ≥15 cfu/plate with the semiquantitative Maki technique or ≥100 cfu/segment with the sonication method.

Skin and hub colonization. Isolation of ≥15 cfu/plate in semiquantitative culture.

NC colonization. Isolation of ≥1 cfu/plate in at least 1 needless connector in the qualitative culture.

C-RBSI. Isolation of the same microorganism both in the colonized PVC tip and in peripheral blood cultures.

### Ethics

The Ethics Committee of our institution (Hospital Gregorio Marañon, number FIBHGM-ECNC 002–2014) approved the study on September 29, 2015. All patients gave their written informed consent to participate.

### Statistical analysis

Regarding the sample size calculation, considering that the rate of phlebitis in PVC was approximately 14% before beginning the study, we estimated that the sample size for the whole study should be 146 patients (a total of 292 catheters in each group, assuming that each patient has an average of two catheters), divided equally in each arm, in order to be able to detect a difference of 7% between the two groups with 80% power and a 5% level of significance.

Qualitative variables are expressed as their frequency distribution. Quantitative variables were expressed as the mean and standard deviation (SD) and as the median and interquartile range (IQR) if the distribution was skewed. Normally distributed continuous variables were compared using the Mann-Whitney test; non-normally distributed continuous variables were compared using the median test. The chi-squared or Fisher exact test was used to compare categorical variables. Statistical significance was set at p≤0.05.

A Kaplan-Meier analysis was conducted in the catheter group in order to assess the risk of phlebitis according to the indwelling time in each group.

Multiple logistic regression analysis was used to assess the risk of tip colonization. The variables included were: days of catheterization, hub colonization, skin colonization, insertion in the emergency department, and intervention group (saline vs. heparin).

We analyzed the data at two different categories: One as “intention to treat”, which corresponds to those patients that were initially included in the study but that, at the end, the catheter tip could not be sent for culture. Therefore, it was imposible to analyze colonization in this group. And other as “by protocol”, which corresponds to those patients of whom we had all the information regarding the catheter, and colonization rate could be assessed.

All other variables independent of catheter information could be analyzed in both groups.

The statistical analysis was performed using SPSS^®^ 21.0.

## Results

### Study population

**Fig 1:**
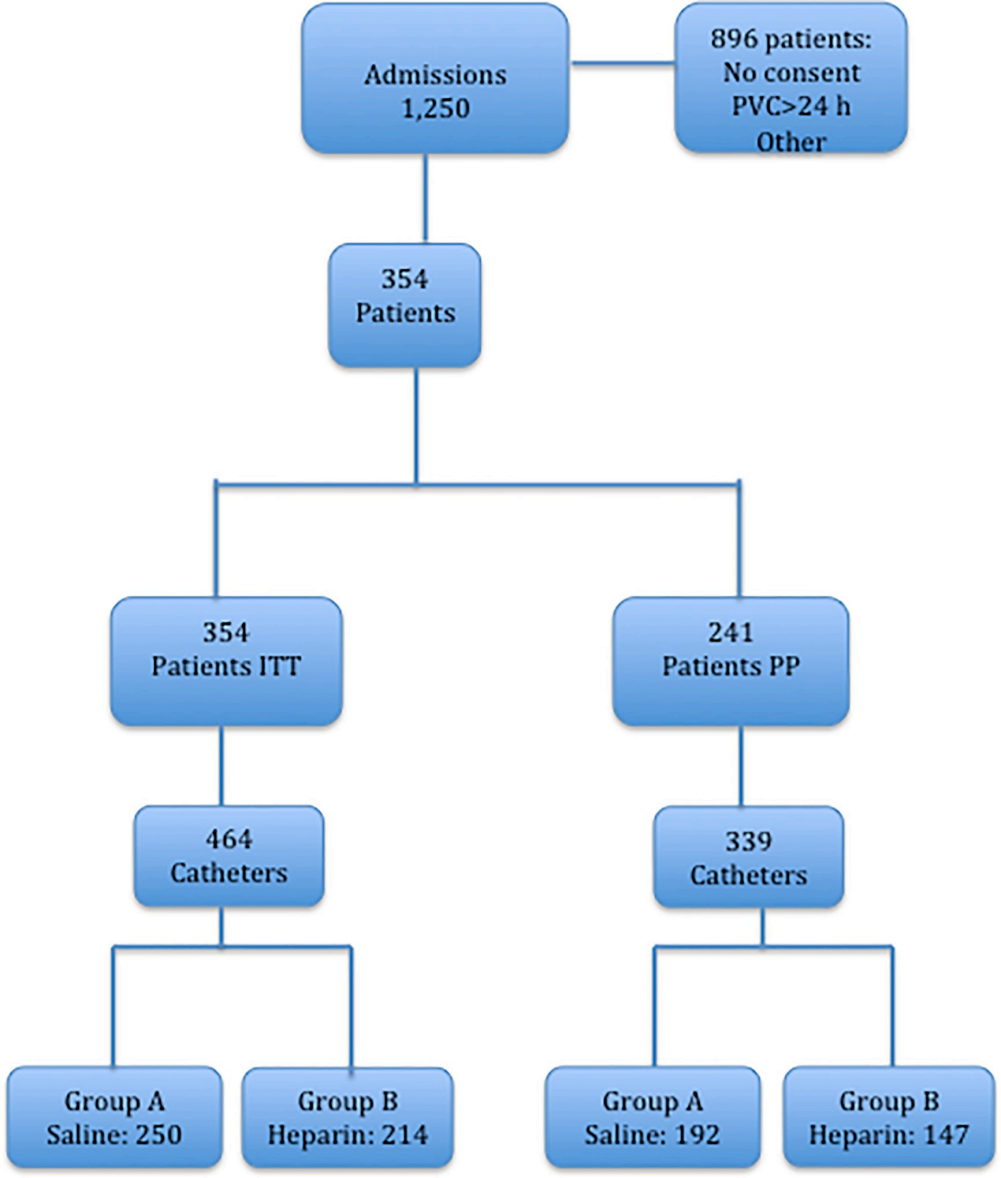
Patient flowchart.

A total of 1,250 patients, were admitted to the IMD during the study period. Of these, 354 (464 catheters) were included in the study: 896 were excluded owing to lack of consent (40%), death, or indwelling time >24 hours (60%), **Fig 1**.

#### Analysis by intention to treat

A total of 464 PVCs from 354 patients were included. Of these, 250 (53.9%) were locked with saline (group A) and 214 (46.1%) were locked with heparin (group B).

**Table 1** describes the characteristics of the study population (354). No significant differences were found between the groups in terms of main demographic characteristics.

**Table 1:** Patients: Analysis of the study population based on intention to treat.

Intention to treat	Total N = 354	SALINE N = 181	HEPARIN N = 173	p
Median (IQR) ^a^ age in years	79.0 (63.1–85.6)	80.5 (65.3–86.5)	78.4 (60.3–85.1)	0.22
Sex, N (%)				0.66
Male	155 (43.8)	77 (42.5)	78 (45.1)	
Female	199 (56.2)	104 (57.5)	95 (54.9)	
Underlying conditions, N (%)				
Myocardial infarction	49 (13.8)	23 (12.7)	26 (15.0)	0.54
Congestive heart failure	139 (39.3)	77 (42.5)	62 (35.8)	0.23
Central nervous system disease	39 (11.0)	21 (11.6)	18 (10.4)	0.73
Chronic obstructive pulmonary disease	96 (27.1)	50 (27.6)	46 (26.6)	0.90
Renal dysfunction	72 (20.3)	39 (21.5)	33 (19.1)	0.59
Diabetes mellitus	94 (26.6)	47 (26.0)	47 (27.2)	0.81
Peptic ulcer disease	28 (7.9)	15 (8.3)	13 (7.5)	0.84
Peripheral vascular disease	22 (6.2)	15 (8.3)	7 (4.0)	0.12
Charlson, median (IQR) ^a^	3 (1–4)	3 (1–4)	2 (1–4)	0.94
Apache II score, median (IQR) ^a^	6 (2.7–6.0)	6 (3.0–6.0)	6 (2.0–6.0)	0.21
Median (IQR) DDDs ^b^	7 (4–129)	7 (4–11.6)	7.8 (4–12)	0.26
Median (IQR) ^a^length of hospital stay in days	7 (5.0–10.0)	7 (5–11.0)	7 (4.0–10.0)	0.33
Median (IQR) ^a^length of IM ^c^ stay in days	7 (4.0–9.0)	6 (4.0–9.5)	7 (4.0–9.0)	0.77
Other infection, N (%)	162 (45.7)	79 (43.6)	83 (48.0)	0.45
Mortality, N (%)	19 (5.4)	14 (7.7)	5 (2.9)	0.06
Episodes of C-RBSI ^d^, N (%)	1 (0.3)	1 (0.6)	0 (0.0)	1.00

^a^ IQR: interquartile range

^b^DDDs: daily defined doses

^c^ IM: internal medicine

^d^ C-RBSI: catheter-related bloodstream infection.

**Table 2** describes the catheters per intention to treat (464). The median (IQR) indwelling time was 5 (3–8) days for both groups (p = 0.64). The frequency of phlebitis in groups A and B was 17.6% and 12.6%, respectively (p = 0.15).

**Table 2:** Catheters per intention to treat.

Catheters	Total N = 464	Saline N = 250	Heparin N = 214	P value
Insertion site, N (%)				
Emergency department	349 (75.2)	182 (72.8)	167 (78.0)	0.19
Location, N (%)				
Hand	33 (7.1)	18 (7.2)	15 (7.0)	1.00
Arm	409 (88.1)	221 (88.4)	188 (87.9)	0.88
Forearm	22 (4.7)	11 (4.4)	11 (5.1)	0.82
Use, N (%)				
Parenteral nutrition	4 (0.8)	2 (0.8)	2 (0.9)	1.00
Fluids	17 (3.7)	10 (4.0)	7 (3.3)	0.80
Antibiotics	296 (63.8)	160 (64.0)	136 (63.6)	0.92
Other	147 (31.7)	78 (31.2)	69 (32.2)	0.84
Reasons for catheter withdrawal, N (%)				
End of use	332	172 (68.8)	160 (74.8)	0.18
Suspicion of infection	71	44 (17.6)	27 (12.6)	0.15
Obstruction	34	23 (9.2)	11 (5.1)	0.10
Other	21	9 (3.6)	12 (5.6)	0.37
Phlebitis, N (%)	71	44 (17.6)	27 (12.6)	0.15
Median (IQR) ^a^ catheter-days	5 (3–8)	5.5 (3–8)	5.0 (3–8)	0.64
Total catheter days	2,903	1,580	1,323	--

^a^ IQR, interquartile range.

No adverse effects were recorded.

### Analysis per protocol

A total of 339 PVCs (241 patients) were finally sent for culture: 192 (56.6%) in the saline group and 147 (43.4%) in the heparin group.

**Table 3** describes the characteristics of the population per protocol (241). The per protocol analysis revealed no significant differences between the groups in terms of the main demographic characteristics.

**Table 3:** Analysis of the study population per protocol.

Per protocol	Total N = 241	SALINE N = 130	HEPARIN N = 111	p
Median (IQR) ^a^ age in years	78.7 (61.3–85.5)	78.7 (61.6–85.3)	78.5 (60.7–85.6)	0.98
Sex, N (%)				0.43
Male	106 (44.0)	54 (41.5)	52 (46.8)	
Female	135 (56.0)	76 (58.5)	59 (53.2)	
Underlying conditions, N (%)	36 (14.9)			
Myocardial infarction	90 (3.7)	16 (12.3)	20 (18.0)	0.27
Congestive heart failure	25 (10.4)	50 (38.5)	40 (36.0)	0.78
Central nervous system disease	73 (30.3)	12 (9.2)	13 (11.7)	0.53
Chronic obstructive pulmonary disease	48 (19.9)	38 (29.2)	34 (30.6)	0.88
Renal dysfunction	65 (27.0)	26 (19.8)	22 (20.0)	1.00
Diabetes mellitus	18 (7.5)	31 (23.8)	34 (30.6)	0.24
Peptic ulcer disease	18 (7.5)	9 (6.9)	9 (8.2)	0.80
Peripheral vascular disease		13 (10.0)	5 (4.5)	0.14
Charlson, median (IQR)^a^	3 (1–4)	2.5 (1–4)	3.0 (1–4)	0.41
Apache II score, median (IQR) ^a^	6 (2–6)	6 (2.7–6)	6 (2–6)	0.92
Median (IQR) ^a^ DDDs ^b^	7 (4–12)	7 (4–11.7)	8 (5–12)	0.25
Median (IQR) ^a^ length of hospital stay in days	7 (5.0–10.0)	7 (5.0–11.0)	7 (5.0–10.0)	0.46
Median (IQR) ^a^ length of IM ^c^ stay in days	7 (5.0–9.0)	7 (.0–10.0)	7 (4.0–9.0)	0.77
Other infection, N (%)	115 (47.7)	59 (45.4)	56 (50.5)	0.44
Mortality, N (%)	12 (4.9)	8 (6.2)	4 (3.6)	0.55
Phlebitis, N (%)	51 (21.2)	30 (23.1)	21 (18.9)	0.26
Episodes of C-RBSI ^d^, N (%)	0 (0.0)	0 (0.0)	0 (0.0)	NA

^a^ IQR: interquartile range

^b^DDDs: daily defined doses

^c^ IM: internal medicine

^d^ C-RBSI: catheter-related bloodstream infection.

**Table 4** describes the catheters per protocol (339). The median (IQR) number of catheter days was 5 (3–8) for both groups (p = 0.54). The rate of phlebitis between the catheters from groups A and B was 21.9% (42/192) and 17.0% (25/147), respectively (p = 0.27). The rate of colonization of PVC tips was 14.6% (28/192) and 12.2% (18/147) in groups A and B, respectively (p = 0.63). There were only 2 episodes of C-RBSI; both occurred in a patient in group A.

**Table 4:** Catheters per protocol.

Catheters	Total N = 339	Saline N = 192	Heparin N = 147	P value
Insertion place, N (%)				
Emergency department	236 (69.6)	131 (68.2)	105 (71.4)	0.55
Location, N (%)				
Hand	31 (9.1)	17 (8.9)	14 (9.5)	0.85
Arm	289 (85.2)	165 (85.9)	124 (84.4)	0.75
Forearm	19 (5.6)	10 (5.2)	9 (6.1)	0.81
Use of catheter, N (%)				
Parenteral nutrition	3 (0.88)	2 (1.0)	1 (0.7)	1.00
Fluids	12 (3.5)	6 (3.1)	6 (4.1)	0.77
Antibiotics	221 (65.2)	129 (67.2)	92 (62.1)	0.42
Medication	103 (30.4)	55 (28.6)	48 (32.7)	0.47
Reasons for catheter withdrawal, N (%)				
End of use	238 (70.2)	126 (65.6)	112 (76.2)	0.04
Suspicion of infection	67 (19.8)	42 (21.9)	25 (17.0)	0.27
Obstruction	31 (9.1)	22 (11.5)	9 (6.1)	0.12
Other	3 (0.9)	2 (1.0)	1 (0.7)	1.00
Median (IQR) ^a^ catheter-days	5 (3–8)	5 (3–8)	5 (3–8)	0.54
Total catheter days	2,019	1,142	877	NA
Phlebitis, N (%)	97 (28.6)	42 (21.9)	25 (17.0)	0.27
Positive cultures, N (%)				
Tip colonization	46 (13.6)	28 (14.6)	18 (12.2)	0.63
Density per 1,000 catheter days	22.8	24.5	20.5	0.66
Skin colonization	64 (18.9)	40 (20.8)	24 (16.3)	0.32
Density per 1,000 catheter days	31.7	35.0	27.4	0.41
Hub colonization	21 (6.2)	15 (7.8)	6 (4.1)	0.12
Density per 1,000 catheter days	10.4	13.1	6.8	0.24
NCT ^b^ colonization	76 (22.4)	41 (21.4)	35 (23.8)	0.60
Density per 1,000 catheter days	37.6	35.9	39.9	0.73
Episodes of C-RBSI ^c^, N (%)	2 (0.6)	2 (1.0)	0 (0.0)	0.50

^a^IQR: interquartile range

^b^NCT: needleless connector

^c^C-RBSI: catheter-related bloodstream infection.

**Fig 2:**
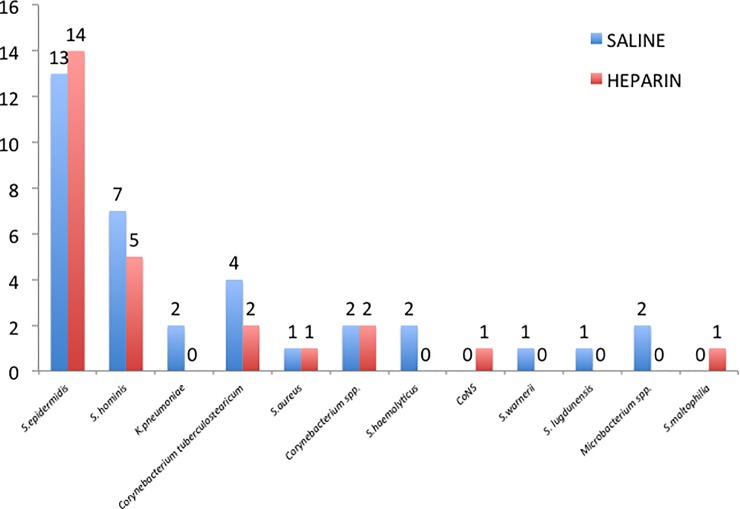
Etiology of the colonized catheters.

We did not find differences between the groups with respect to the etiology of colonization (**Fig 2**).

The skin was colonized in 22% of the 236 PVCs inserted in the emergency department (p = 0.01).

**Table 5** describes multivariate logistic regression analysis of tip colonization. Logistic regression analysis revealed colonization of skin and hubs as the only independent risk factors for catheter tip colonization.

**Table 5:** Multivariate logistic regression analysis of tip colonization.

	Odds ratio	β	p-value	95% CI
Insertion in emergency department	1.24	0.21	0.72	0.37–4.14
Group (saline/heparin)	1.51	0.41	0.40	0.56–4.02
Skin colonization	92.26	4.52	<0.001	28.35–300.18
Hub colonization	25.97	3.25	<0.001	4.63–145,7
Catheter days	1.04	0.04	0.54	0.90–1.20

**Fig 3:**
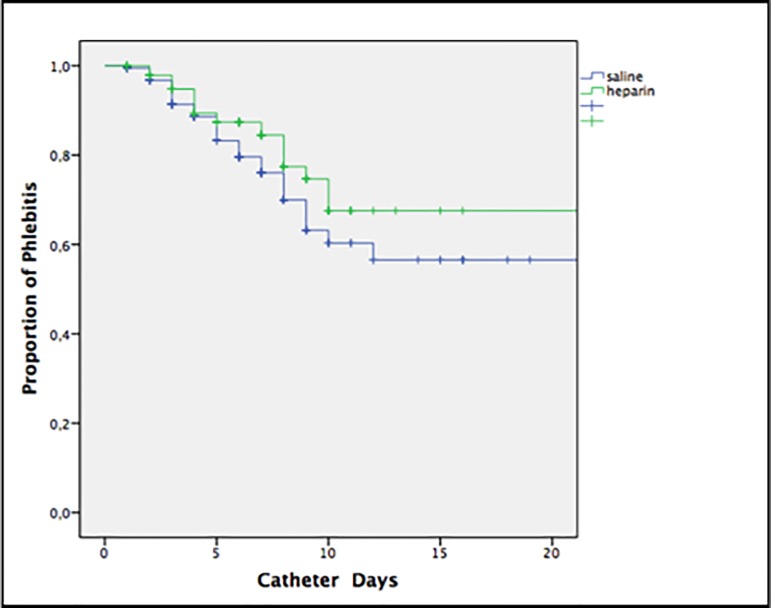
Kaplan-Meier analysis of the risk of phlebitis according to catheter days.

The Kaplan-Meier analysis did not reveal statistically significant differences for phlebitis according to the days of catheter in each group (long-rank test p = 0.19), **Fig 3**.

## Discussion

We found no significant differences regarding phlebitis and catheter tip colonization rates in PVCs locked either with saline or heparin.

The increasing use of PVCs results in a rate of infection similar to that of central venous catheters when these catheters are monitored [5,6]. However, recommended preventive measures, such as using heparin locks for catheter maintenance, are not as rigorous as those recommended for central venous catheters [17,21]. This issue remains controversial because of the lack of randomized controlled trials and the heterogeneous results reported [10,22].

We proved that is not necessary to lock PVCs with heparin, as phlebitis and catheter tip colonization rates were similar for PVCs locked with saline and PVCs locked with heparin. Our findings are similar to those of other studies comparing saline and heparin in the maintenance of PVCs: we found no statistically significant differences between the groups regarding catheter obstruction rate, indwelling time, phlebitis, and accidental catheter removal [22] [10,16]. On the other hand, indwelling time has been reported to be longer in PVCs locked with heparin [23]. A systematic review and meta-analysis examining catheter maintenance showed that continuous infusion of heparin in PVCs improved the duration of patency and reduced infusion failure and phlebitis. However, no statistically significant differences were found when heparin was used intermittently, which is the usual protocol for catheter locking [22].

Our study revealed the frequency of phlebitis to be 28.6%, which was similar to percentages reported elsewhere (9%-33%) [7,24,25].

In addition, our data showed that catheter insertion in the emergency department was associated with high skin colonization and phlebitis rates, as reported elsewhere [26,27]. Therefore, when it is not possible to ensure adherence to guidelines by the personnel inserting catheters in the emergency room, catheters should be replaced within 48 hours.

Despite it was no statistical significance, we found a difference between mortality rate in both groups. However, we consider that it may have no impact in the study endpoints. We also had very difficult to include patients in this study because many patients came from the emergency room with the peripheral venous catheter inserted more than 24 hours.

Our study is subject to limitations, and our results must be interpreted with caution. We were unable to identify the number of flushes performed for each catheter or to assess whether the nurses who inserted the catheter had received appropriate training. In addition, since we only enrolled patients from the IMD, our data cannot necessarily be extrapolated to other populations.

Another limitation was that, despite the tip could not be collected in many patients for different reasons and it reduce the sample size, we did not find significant differences between both groups.

While it is recommended to use a 10-mL diameter syringe and prefilled flush syringes, flushing practices for PVCs appear to vary widely. Therefore, we consider it is necessary to perform future studies to standardize volumes and frequency of flushing for the maintenance of PVCs [12,14,15,28].

To conclude, we consider that PVCs can be locked with saline for maintenance, as this is safer and cheaper than heparin.
